# Phenotypic characteristics of colo-rectal cancer in I1307K APC germline mutation carriers compared with sporadic cases

**DOI:** 10.1054/bjoc.2001.2093

**Published:** 2001-09-01

**Authors:** A Figer, R Shtoyerman-Chen, A Tamir, R Geva, L Irmin, D Flex, L Theodor, A Sulkes, S Sadetzki, S Bar-Meir, E Friedman

**Affiliations:** Institute of Oncology Rabin Medical Center, Beilinson Campus, Petach-Tikvah; Department of Gastroenterology; Cancer Epidemiology Unit, Gertner Institute of Public Health; Susanne-Levy Gertner Oncogenetics Unit, Danek Gertner Institute of Genetics, Sheba Medical Center, Tel-Hashomer, 52621 and the Sackler School of Medicine, Tel-Aviv University, Ramat Aviv, Israel; Department of Epidemiology, Carmel Medical Center, Haifa, Israel

**Keywords:** inherited predisposition to cancer, I1307K APC mutation, phenotype

## Abstract

The I1307K APC germline mutation is associated with an increased risk to colo-rectal cancer (CRC). Whether and to what extent the phenotype of CRC in mutation carriers differs from sporadic cases, remains unknown. To gain insight into this issue, we analysed 307 unselected Israeli patients with CRC, who were treated in a single medical centre, for harbouring the I1307K mutation. Twenty-eight mutation carriers (9.1%) were detected. Two of 28 mutation carriers (7.1%) and 93/277 (33.6%) of non-carriers, were of non-Ashkenazi origin (*P*< 0.01). In 74/278 (26.6%) of the sporadic cases, and only 1/28 (3.6%) of mutation carriers (3.6%) the tumour was located in the right colon (*P*< 0.01). Mutation carriers had a more advanced disease stage (14/28 – 50% Dukes C), as compared with 60 (19.5%) of non-carriers (*P*= 0.02). The mean age at diagnosis was similar: 65 (+/– 9.7) years and 66.3 (+/– 11.6) years, for mutation carriers and non-carriers, respectively. No statistical differences were noted between the two groups in sex distribution, tumour grade, and family history of cancer. We conclude that early age at diagnosis and family history of cancer cannot be used to predict who is likely to harbour the I1307K APC germline mutation carriers. However, the tumours in patients with this mutation appear different than those without, are less likely to be proximal and more likely to be advanced than tumours in non-carriers.   http://www.bjcancer.com © 2001 Cancer Research Campaign


*British Journal of Cancer* (2001) **85**(9), 1368–1371
© 2001 Cancer Research Campaign
doi: 10.1054/ bjoc.2001.2093, available online at http://www.idealibrary.com on IDEAL

http://www.bjcancer.com

### Phenotypic characteristics of colo-rectal cancer in I1307K APC germline mutation carriers compared with sporadic cases

**A Figer[1], R Shtoyerman-Chen[2], A Tamir[5], R Geva[1], L Irmin[1,4], D Flex[1], L Theodor[2], A Sulkes[1], S Sadetzki[3], S Bar-Meir[2] and E Friedman[4]**

[1]Institute of Oncology Rabin Medical Center, Beilinson Campus, Petach-Tikvah;[2] Department of Gastroenterology; [3]Cancer Epidemiology Unit, Gertner Institute of Public Health;[4] Susanne-Levy Gertner Oncogenetics Unit, Danek Gertner Institute of Genetics, Sheba Medical Center, Tel-Hashomer, 52621 and the Sackler School of Medicine, Tel-Aviv University, Ramat Aviv, Israel; and [5]Department of Epidemiology, Carmel Medical Center, Haifa, Israel

**Summary** The I1307K APC germline mutation is associated with an increased risk to colo-rectal cancer (CRC). Whether and to what extent the phenotype of CRC in mutation carriers differs from sporadic cases, remains unknown. To gain insight into this issue, we analysed 307 unselected Israeli patients with CRC, who were treated in a single medical centre, for harbouring the I1307K mutation. Twenty-eight mutation carriers (9.1%) were detected. Two of 28 mutation carriers (7.1%) and 93/277 (33.6%) of non-carriers, were of non-Ashkenazi origin ($P < 0.01$). In 74/278 (26.6%) of the sporadic cases, and only 1/28 (3.6%) of mutation carriers (3.6%) the tumour was located in the right colon ($P < 0.01$). Mutation carriers had a more advanced disease stage (14/28 – 50% Dukes C), as compared with 60 (19.5%) of non-carriers ($P = 0.02$). The mean age at diagnosis was similar: 65 (+/– 9.7) years and 66.3 (+/– 11.6) years, for mutation carriers and non-carriers, respectively. No statistical differences were noted between the two groups in sex distribution, tumour grade, and family history of cancer. We conclude that early age at diagnosis and family history of cancer cannot be used to predict who is likely to harbour the I1307K APC germline mutation carriers. However, the tumours in patients with this mutation appear different than those without, are less likely to be proximal and more likely to be advanced than tumours in non-carriers. © 2001 Cancer Research Campaign http://www.bjcancer.com

**Keywords**: inherited predisposition to cancer; I1307K APC mutation; phenotype

A missense mutation within the *APC* gene, Isoleucine to Lysine alteration in codon 1307 (I1307K), has been described in Jewish individuals of East European descent (Ashkenazi), both at risk for colorectal cancer (CRC), and also in the general, average risk, population of the same ethnic extraction (Laken et al, 1997; Woodage et al, 1998). Studies involving I1307K carriers have shown a moderate increase (up to 2-fold) in colon cancer risk (Laken et al, 1997; Woodage et al, 1998; Rozen et al, 1999; Gryfe et al, 1999). This specific polymorphism was considered to be limited to Ashkenazi Jews (Laken et al, 1997; Prior et al, 1999), but it was also reported in Jewish individuals of non-Ashkenazi origin (Rozen et al, 1999; Patael et al, 1999; Drucker et al, 2000; Shtoyerman et al, 2001), as well as non-Jewish individuals (Yuan et al, 1998; Nathanson et al, 1999). Yet, the rate of this mutation in the Jewish Ashkenazi population (5–7%) (Laken et al, 1997; Woodage et al, 1998) and among patients with familial CRC (up to 28%) (Laken et al, 1997) facilitates large-scale analysis of this mutation in unselected Jewish sub-populations. While most studies tend to support the role this missense mutation plays in conferring an increased risk to CRC (Woodage et al, 1998; Gryfe et al, 1999), little information exists as to the phenotypic features of mutation-associated CRC. Some studies claimed that mutation carriers have an earlier age at onset of CRC as compared with the general population (Laken et al, 1997; Gryfe et al, 1999), or an association with multiple colonic adenomas (Gryfe et al, 1999). We determined the phenotypic features of 307 unselected CRC patients who were treated in a single medical centre in Israel, and compared these features in I1307K mutation carriers with non-carriers.

*Received 15 May 2001*
*Revised 1 August 2001*
*Accepted 7 August 2001*

*Correspondence to:* E Friedman

### MATERIALS AND METHODS

### Patients

All analysed patients had clinical and pathological confirmation of CRC, and were being treated and followed-up at the Oncology Institute at the Rabin Medical Center (Belinson campus). The institutional review board approved the study, and each participant signed a written informed consent. All clinical details were retrieved by a detailed questionnaire filled by the patients, and extraction of data from the medical records and pathology reports. Patients who fulfilled the Amsterdam or Bethesda II criteria for HNPCC (OMIM # 114500) (OMIM database), were excluded from analysis.

### DNA extraction

Genomic DNA was prepared from anticoagulated venous blood samples using standard techniques, employing the Gentra Kit (Gentra Inc., Minneapolis, MN) and using the manufacturer's recommended protocol.

### Analysis of the I1307K mutation

I1307K mutation detection included PCR amplification of the relevant genomic region, and subjecting the resulting PCR product to analysis by denaturing gradient gel electrophoresis (DGGE) as detailed elsewhere (Patael et al, 1999), or a modified restriction analysis (MRA), as detailed elsewhere by us (Shtoyerman et al, 2001). All consistently abnormally migrating fragments (i.e., repeated abnormalities on three independent PCRs) were subject to sequence analysis using the big Dye terminator chemistry and kit (PE Biosystems, Foster City, CA), and using the ABI Prism 310 semiautomatic DNA sequencer (PE Biosystems).

### Statistical analysis

Statistical analysis was performed using the Chi square test or the Fisher Exact test where appropriate. Mann-Whitney test was used to compare ordinal variables between the two groups. When a non-statistically significant result was obtained, power calculations were performed. These calculations were based on the estimated parameters among carriers, at effect size deemed to be biologically important, using the same sample size.

### RESULTS

### Overall patients' characteristics

Overall, 307 patients were analysed: 170 men and 137 women. There were 189 patients of Ashkenazi origin (61.6%), 80 non-Ashkenazis (26%) (30 of Iraqi origin, 21 of North African origin, 11 of Balkan origin, and 18 of Yemenite extraction). There were 22 mixed Israeli-Ashkenazis, 14 Mixed Israeli non-Ashkenazis, and 2 Israeli Arabs. A total of 118 patients (38.4%) had first degree relatives with cancer: 55 (17.9%) had relatives with CRC, 53 with non-gastrointestinal tract tumours, and in 10 patients – other malignancies that were not specified were noted in relatives. Disease stage in the majority of patients (188 – 61.2%) was Dukes B, and in 74 (24.1%) Dukes C. The majority of tumours were either well differentiated (66 – 21.5%) or moderately differentiated (178 – 58%). Tumours were most commonly located at the rectosigmoid region ($n$ = 172 – 56%), and in 74 (24.2%) the tumour was located in the right colon.

### Comparison of the I1307K mutation carriers with non-carriers

There were 28 (9.1%) I1307K mutation carriers, and the rest were non-carriers. The age at diagnosis, sex, ethnic origin, disease grade and stage, tumour location and family history of cancer in carriers compared with non-carriers is shown in Table 1. As is evident from the table, mean age at diagnosis of carriers (65.0 +/– 9.7 years) is not significantly different than that of non-carriers (66.3 +/– 11.6 years). Assuming that carriers are 5 years younger than non-carriers, our sample gave a power that was equal to 0.88. The features that statistically differed in mutation carriers and non-carriers are the predicted paucity of non-Ashkenazis among mutation carriers (2–7.1%) compared with the rate among non-carriers (93 – 33.6%) ($P < 0.01$). In 74 (26.6%) of the sporadic cases and only one mutation carrier (3.6%), the tumour was located in the right colon ($P < 0.01$), and in 15 mutation carriers (53.6%) disease stage was Dukes C or higher (53.6%) compared with 80 (28%) of non carriers ($P = 0.02$). For the parameters that were not significantly different between carriers and non-carriers, power calculations were carried out. To detect a two-fold difference in positive family history of colon or other cancer in carriers and a two fold increase in the proportion of more advanced disease (poorly and undifferentiated) in carriers our sample size was clearly inadequate, giving power of 0.33 and 0.15, respectively.

### DISCUSSION

In this study, most of the phenotypic features of CRC in I1307K mutation carrier did not markedly differ from those of non-mutation carriers. Notably, age at diagnosis and the rate of a positive family history of cancer were similar in carriers and non-carriers. This finding is counter-intuitive, as the prevailing paradigm of cancer susceptibility genes predicts that mutation carriers will exhibit early age at onset, and have a personal or a family history of cancer more frequently than sporadic cases. The findings of a similar age at onset and family history of cancer in mutation carriers and non-carriers reported herein are consistent with another study (Drucker et al, 2000). Taken together with the

**Table 1** Comparison of the phenotypic features of I1307K mutation carriers as compared with non-carriers

| Parameter | I1307K carriers ($n$ = 28) (%) | Non-carriers ($n$ = 279) (%) | $P$ value |
|---|---|---|---|
| Age at diagnosis | | | NS |
| Range | 38–83 | 26–89 | |
| Mean +/– SD | 65.0 ± 9.7 | 66.3 ± 11.6 | |
| Sex | | | NS |
| Males | 12 (42.9) | 158 (56.6) | |
| Females | 16 | 121 | |
| Ethnic origin | | | |
| Ashkenazis | 23 (92.3)* | 166 (66.3)* | |
| Non-Ashkenazis (Total) | 2 (7.1) | 93 (33.6) | 0.01 |
| Asia | 1 | 29 | |
| North Africa | | 21 | |
| Balkan | | 11 | |
| Yemen | | 18 | |
| Mixed Ashkenazis | 3 | 19 | |
| Mixed non-Ashkenazis | 1 | 13 | |
| Arab-Israelis | | 2 | |
| Family history of Cancer | | | NS |
| Colon | 5 | 50 | |
| Other | 5 | 58 | |
| Tumour Location | | | 0.01 |
| Right colon | 1 (3.6) | 74 (26.6)** | |
| Transverse | 7 (25) | 20 (7.2) | |
| Left colon | 8 (28.6) | 110 (39.7) | |
| Rectum | 12 (42.9) | 73 (26.4) | |
| Tumour grade | | | NS |
| Well Differ | 6 (21.4) | 60 (21.5) | |
| Moderate Differ | 17 (60.7) | 161 (57.7) | |
| Poorly Differ | 2 (7.1) | 21 (7.5) | |
| Undifferentiated | – | 1 (0.4) | |
| Unknown | 3 (10.7) | 36 (12.9) | |
| Disease Stage | | | 0.02*** |
| Dukes A | – | 12 (4.3) | |
| Dukes B | 13 (46.4) | 175 (62.7) | |
| Dukes C | 14 (50) | 60 (21.5) | |
| Distant metastases | 1 (3.6) | 20 (7.2) | |
| Unrecorded | – | 12 (4.3) | |

*Includes the Mixed Ashkenazis; **For two non-carriers, the tumour location was unrecorded; ***Mann Whitney test applied.

© 2001 Cancer Research Campaign 

reports that adenomatous colonic polyps were not more prevalent in I1307K mutation carriers compared with non-carriers (Syngal et al, 2000; Stern et al, 2001), these findings imply that the I1307K APC mutation confers a moderate increased lifetime risk for CRC (Woodage et al, 1998; Gryfe et al, 1999). Yet, the classical phenotypic parameters used for identifying individuals at risk for harbouring a germline mutation in cancer susceptibility gene may not be directly applied for the I1307K APC mutation. Thus, this missense mutation may prove to be one of several ‘functional polymorphisms’, each with a moderate effect of increasing cancer risk, with the combined effect in carriers of many of these polymorphisms, manifesting as CRC.

The I1307K mutation was originally described among Ashkenazi Jews (Laken et al, 1997), where it occurs at a rate of up to 7% of the general population (Woodage et al, 1998). However, this mutation was reported at a significantly lower rate in non-Ashkenazi Jews (Rozen et al, 1999; Patael et al, 1998) and anecdotally in non-Jewish individuals (Yuan et al, 1998; Nathanson et al, 1999). In fact, we previously showed that the mutation probably represents a founder mutation in Jewish individuals (Patael et al, 1998), as was also confirmed by other investigators (Woodage et al, 1998). Drucker et al (2000), reported that the I1307K mutation was detected in 3/4 Yemenite Jewish individuals, whereas in the present study this mutation could not be detected in any of 18 Yemenite Jews with CRC. This discrepancy cannot be logically settled, until more individuals of Yemenite extraction are tested. The age standardized rate (ASR) of CRC in Ashkenazis in Israel is 41.9/100 000 and in non-Ashkenazis, including Yemenites–25.5/100 000 (Israel Cancer Registry, 1998). This reality of CRC epidemiology in Israel intuitively suggests that the rate of this missense mutation is lower in Yemenites than in Ashkenazis. If indeed this mutation contributes to CRC pathogenesis in the general population, than one must assume that modifier factors, such as environmental exposures, personal habits, nutritional factors and modifier genes affect the phenotypic expression of this mutation in Yemenite Jews.

Mutation carriers had a more advanced disease stage, in line with the observations from another study from Israel (Drucker et al, 2000). Yet, several differences exist between the data presented herein and the study by Drucker and co-workers (2000). In that study, tumours in mutation carriers tended to cluster to the right colon and have a more favourable histological grade, whereas neither was observed in our study population. Moreover, more than 40% of mutation carriers displayed rectal cancer, a high rate compared with non-carriers in this study or in unselected series of colo-rectal cancer patients in Israel (Israel Cancer Registry, 1998). One possible explanation is that there are differences in the selection criteria between the two medical centres. This seems unlikely, as both medical centres are located in the same geographical region of Israel, and both attract the same ethnic profile of patients. Another possibility is that the small number of analysed patients in both studies combined prevent firm conclusions as to the putative role that the I1307K plays in affecting the phenotypic features of CRC. The published studies that addressed the issue of the phenotypic features of colon cancer in I1307K APC mutation carriers are summarized in Table 2. The inconclusive and at times conflicting results from these studies indicate that a much larger study, encompassing a large number of individuals, with a longer follow-up is urgently needed.

The notion that the phenotype of CRC in I1307K mutation carriers is for the most part similar to that of non-carriers, is indirectly supported by the study of Syngal and co-workers (2000). In that study, the phenotypic features of adenomatous polyps, as well as the family history of cancer, did not differ in mutation carriers as compared with non-carriers.

**Table 2** Comparisons of the results of this study with previously published studies on the phenotypic features of colon cancer in I1307K mutation carriers

| | Present study | Gryfe (1999) | Drucker (2000) | Stern (2001) |
|---|---|---|---|---|
| Total number of CRC patients analysed | 307 | 404 | 111 | 22 |
| Number of carriers (%) | 28 (9.0) | 41 (10.1) | 15 (13.5) | 6 (27.3) |
| Age at diagnosis (Mean + SD) | 65.0 ± 9.7 | 70.2 ± 1.2 | 64.6 ± 10 | NA* |
| Family history of Colon Cancer (%) | 5 (17.8) | NA | NA | 3 (50) |
| Dukes stage C and higher (%) | 15 (53.5) | NA | 11 (73.3) | NA |
| Grade | | | | |
| Well/moderate differentiated | 23 | NA | 15 | NA |
| Poorly differentiated | 2 | NA | 0 | NA |
| Tumour location | | | | |
| Right | 1 | 13*** | 12 | NA |
| Transverse | 7 | | | |
| Left | 8 | 20** | 2** | |
| Rectum | 12 | 14 | 1 | |
| Associated polyps | NA | 1.92 ± 0.08 | NA | ND**** |

*NA Denotes data not available; **Includes both transverse and left colon; ***In Gryfe et al (1999) the total number of tumours includes seven patients with adenomas; ****No difference between carriers and non-carriers.

In conclusion, early age at diagnosis or family history of CRC or right-sided tumour are poor predictors of detecting the I1307K APC mutation, and with the possible exception of a more advanced disease stage, the phenotypic features of CRC are similar in mutation carriers and non-carriers.

### ACKNOWLEDGEMENT

This study was funded in part by a generous donation from Mr Ami Yaar in loving memory of his wife, Ruti.

### REFERENCES

Drucker L, Shpilberg O, Neumann A, Shapira J, Stackievicz R, Beyth Y and Yarkoni S (2000) Adenomatous polyposis coli I1307K mutation in Jewish patients with different ethnicity: prevalence and phenotype. *Cancer* **88**: 755–760

Gryfe R, Di Nicola N, Lal G, Gallinger S and Redston M (1999) Inherited colorectal polyposis and cancer risk of the APC I1307K polymorphism. *Am J Hum Genet* **64**: 378–384

Israel Cancer Registry (1998) *Cancer in Israel: facts and figures 1992–1995*. Jerusalem

Laken SJ, Petersen GM, Gruber SB Oddoux C, Ostrer H, Giardiello FM, Hamilton SR, Hampel H, Markowitz A, Klimstra D, Jhanwar S, Winawer S, Offit K, Luce MC, Kinzler KW and Vogelstein B (1997) Familial colorectal cancer in Ashkenazim due to a hypermutable tract in *APC. Nat Genet* **17**: 79–83

Nathanson KL, Antin-Ozerkis D, Couch FJ and Weber BL (1999) I1307K APC variant in non-Ashkenazi Jewish women affected with breast cancer. *Am J Med Genet* **85**: 189–190

OMIM database – http://www.ncbi.nlm.nih.gov/htbin-post/Omim/dispmim?114500

Patael Y, Figer A, Gershoni-Baruch R, Papa MZ, Risel S, Chen R, Karasik A, Theodor L and Friedman E (1999) A common origin of the I1307K APC polymorphism in Ashkenazi and non-Ashkenazi Jews. *Eur J Hum Genet* **7**: 555–559

 *© 2001 Cancer Research Campaign*

Prior TW, Chadwick RB, Papp AC, Arcot AN, Isa AM, Pearl DK, Stemmermann G, Percesepe A, Loulka A, Aaltonen LA and De La Chapelle A (1999) The I1307K polymorphism of the *APC* gene in colorectal cancer. *Gastroenterology* **116**: 58–63

Rozen P, Shomrat R, Strul H Naiman T, Karminsky N, Legum C and Orr-Urtreger A (1999) Prevalence of the I1307K *APC* gene variant in Israeli Jews of differing ethnic origin and risk for colorectal cancer. *Gastroenterology* **116**: 54–57

Shtoyerman-Chen R, Friedman E, Figer A, Carmel M, Patael Y, Rath P, Fidder HH, Bar-Meir S and Theodor L (2001) The I1307K APC Polymorphism: Prevalence in non-Ashkenazi Jews and Evidence for a Founder Effect. *Genetic Testing* **5(2)**: 141–146

Stern HS, Viertelhausen S, Hunter AGW, O'rourke K, Cappelli M, Perras H, Serfas K, Blumenthall A, Dewar D, Baumann E and Lagarde AE (2001) APC I1307K increases risk of transition from polyp to colorectal carcinoma in Ashkenazi Jews. *Gastroenterology* **120**: 392–400

Syngal S, Schrag D, Falchuk M, Tung N, Farraye FA, Chung D, Wright M, Whetsell A, Miller G and Garber JE (2000) Phenotypic characteristics associated with the APC gene I1307K mutation in Ashkenazi Jewish patients with colorectal polyps. *JAMA* **284**: 857–860

Woodage T, King SM, Wacholder S, Hartge P, Struewing JP, McAdams M, Laken SJ, Tucker MA and Brody LC (1998) The APCI1307K allele and cancer risk in a community-based study of Ashkenazi Jews. *Nat Genet* **20**: 62–65

Yuan ZQ, Kasprzak L, Gordon PH, Pinsky L and Foulkes WD (1998) I1307K APC and hMLH1 mutations in a non-Jewish family with hereditary non-polyposis colorectal cancer. *Clin Genet* **54**: 368–370

*© 2001 Cancer Research Campaign*
